# Generation of lentivirus-induced dendritic cells under GMP-compliant conditions for adaptive immune reconstitution against cytomegalovirus after stem cell transplantation

**DOI:** 10.1186/s12967-015-0599-5

**Published:** 2015-07-22

**Authors:** Bala Sai Sundarasetty, Stephan Kloess, Olaf Oberschmidt, Sonja Naundorf, Klaus Kuehlcke, Anusara Daenthanasanmak, Laura Gerasch, Constanca Figueiredo, Rainer Blasczyk, Eliana Ruggiero, Raffaele Fronza, Manfred Schmidt, Christof von Kalle, Michael Rothe, Arnold Ganser, Ulrike Koehl, Renata Stripecke

**Affiliations:** REBIRTH, Regenerative Immune Therapies Applied, Hannover Medical School, OE6862, Hans Borst Zentrum, Carl Neuberg Strasse 1, 30625 Hannover, Germany; Department of Hematology, Hemostasis, Oncology and Stem Cell Transplantation, Hannover Medical School, OE6862, Hans Borst Zentrum, Carl Neuberg Strasse 1, 30625 Hannover, Germany; EUFETS GmbH, Idar-Oberstein, Germany; Institute of Cellular Therapeutics and GMP Core Facility IFB-Tx, Hannover Medical School, Hannover, Germany; REBIRTH, Tolerogenic Cell Therapy, Department of Transfusion Medicine, Hannover Medical School, Hannover, Germany; Division of Translational Oncology, National Center for Tumor Diseases, Heidelberg, Germany; Institute of Experimental Hematology, Hannover Medical School, Hannover, Germany

**Keywords:** Monocyte, Dendritic cell, Lentiviral vector, Stem cell transplantation, Cytomegalovirus

## Abstract

**Background:**

Reactivation of latent viruses such as human cytomegalovirus (HCMV) after allogeneic hematopoietic stem cell transplantation (HSCT) results in high morbidity and mortality. Effective immunization against HCMV shortly after allo-HSCT is an unmet clinical need due to delayed adaptive T cell development. Donor-derived dendritic cells (DCs) have a critical participation in stimulation of naïve T cells and immune reconstitution, and therefore adoptive DC therapy could be used to protect patients after HSCT. However, previous methods for ex vivo generation of adoptive donor-derived DCs were complex and inconsistent, particularly regarding cell viability and potency after thawing. We have previously demonstrated in humanized mouse models of HSCT the proof-of-concept of a novel modality of lentivirus-induced DCs (“SmyleDCpp65”) that accelerated antigen-specific T cell development.

**Methods:**

Here we demonstrate the feasibility of good manufacturing practices (GMP) for production of donor-derived DCs consisting of monocytes from peripheral blood transduced with an integrase-defective lentiviral vector (IDLV, co-expressing GM-CSF, IFN-α and the cytomegalovirus antigen pp65) that were cryopreserved and thawed.

**Results:**

Upscaling and standardized production of one lot of IDLV and three lots of SmyleDCpp65 under GMP-compliant conditions were feasible. Analytical parameters for quality control of SmyleDCpp65 identity after thawing and potency after culture were defined. Cell recovery, uniformity, efficacy of gene transfer, purity and viability were high and consistent. SmyleDCpp65 showed only residual and polyclonal IDLV integration, unbiased to proto-oncogenic hot-spots. Stimulation of autologous T cells by GMP-grade SmyleDCpp65 was validated.

**Conclusion:**

These results underscore further developments of this individualized donor-derived cell vaccine to accelerate immune reconstitution against HCMV after HSCT in clinical trials.

**Electronic supplementary material:**

The online version of this article (doi:10.1186/s12967-015-0599-5) contains supplementary material, which is available to authorized users.

## Zusammenfassung

**Hintergrund:**

Die Reaktivierung latenter Viren wie das humane Cytomegalovirus (HCMV) führt zu einer hohen Morbidität und Mortalität nach allogener Stammzelltransplantation (allo-HSZT). Aufgrund verzögerter T-Zell-Entwicklung nach allo-HSZT ist eine wirksame Immunisierung der Patienten gegen HCMV von großer klinischer Bedeutung. Dabei spielt die Immunrekonstitution Dendritischer Zellen (DCs) eine wichtige Rolle. Frühere Verfahren zur ex vivo Generierung von DCs zur klinischen Anwendung sind komplex und wenig reproduzierbar, insbesondere im Hinblick auf die Vitalität und Potenz der Zellen nach der Kryopreservierung. In früheren Arbeiten konnten wir in humanisierten Stammzelltransplantations-Maus-Modellen eine neue Methode mittels Lentivirus-induzierten DCs (“SmyleDCpp65”) vorstellen, die zu einer beschleunigten Entwicklung antigen-spezifischer T-Zellen führt.

**Verfahren:**

In der vorliegenden Arbeit zeigen wir die Möglichkeit, Monozyten mit einem Integrase-defekten lentiviralen Vektor (IDLV) unter guter Herstellungspraxis (GMP) zu transduzieren zur Ko-expression von GM-CSF, IFN-α und pp65 Zytomegalovirus Antigen. Nach Transduktion wurden die Zellen kryokonserviert.

**Ergebnisse:**

Die standardisierte Produktion des IDLVs und die Herstellung von SmyleDCpp65 (n=3) unter GMP-konformen Bedingungen konnte demonstriert werden. Analytische Parameter zur Qualitätskontrolle der SmyleDCpp65 Identität nach dem Auftauen und Potenz nach der Kultivierung wurden definiert. Zellgewinnung, Uniformität der Zellen, Effizienz des Gentransfers, Reinheit und Vitalität waren hoch und konsistent. SmyleDCpp65 Zellen zeigten geringe IDLV Integrationen im Genom und ein polyklonales Integrationsmuster ohne Präferenz zu Protoonkogenen. Letztendlich wurde ein Verfahren zur Stimulation autologer T-Zellen durch GMP-SmyleDCpp65 validiert.

**Fazit:**

Die weitere Entwicklung dieser individuellen Zellvakzine für klinische Studien ist von hoher Relevanz, um die Immunrekonstitution gegen Zytomegalovirus nach allo-HSZT zu beschleunigen.

## Background

Allogeneic hematopoietic stem cell transplantation (allo-HSCT) is a routine standard of care procedure for preventing relapse in patients with hematologic malignancies such as acute myeloid leukemia (AML) [1]. Cytoreductive conditioning regimens, T cell depletion and immune suppressive therapies used in the context of allo-HSCT elicit a delay in adaptive immunity, predisposing patients to infections. Regeneration of naïve and memory T cells after allo-HSCT requires the de novo production of naïve T cells in thymus and memory T cells in the periphery [2]. Among infections and reactivations after allo-HSCT, human cytomegalovirus (HCMV) is a major challenge for clinicians and patients due to high morbidity, mortality and significant costs for management with antiviral drugs or adaptive T cell therapy [3, 4]. No effective clinical vaccines are currently approved against HCMV in the allo-HSCT setting [5]. At the time when the patients need immunological protection the most, they are still severely lymphopenic, immune compromised or immune suppressed and the state-of-the-art vaccines do not provide effective protective immunity. Third party adoptive T cells require complex manufacturing and are in later phase clinical trials (NCT01077908), but have not received approval or pricing authorization by the FDA or EMA yet. Thus, simple innovative, relatively inexpensive and individualized cell therapy approaches are warranted to cover this unmet clinical need.

Dendritic cells (DCs) are potent regulators of immunity capable of priming naïve lymphocytes for long-lasting and highly efficient adaptive immune responses. One special hallmark the immune surveillance by DCs is their migratory behavior from tissues to lymph nodes (LN), where they utilize the optimized cyto-architecture in germinal centers to encounter and stimulate naïve T and B cells. T cell receptors (TCRs) are stimulated by specific antigenic epitopes presented by the major histocompatibility complex (MHC) highly expressed on DCs [6]. DCs have different mechanisms to internalize and process antigens, and a long-lasting exposure of naïve T and B cells to antigens processed and presented by DCs in LN can maximize the immunologic synapse for selection of high-affinity TCRs and B cell receptors (BCRs) [7].

Even up to 1 year after allo-HSCT, DC levels are usually abnormal and patients with faster DC recovery show lower mortality [8]. Incidentally, HCMV can further hamper immune reconstitution by interfering with dendritic cell differentiation and function [9]. Thus, the use of adoptive ex vivo “conventional” monocyte-derived DC has been explored in a few phase I/II studies to protect or treat HSCT recipients against HCMV [10, 11]. In one study, patients immunized with peptide-loaded donor-derived conventional DCs showed a proof-of-concept clinical benefit with induction of HCMV-specific cytotoxic T lymphocytes (CTLs) in 30% of patients [11]. Notably, this pilot clinical trial demonstrated no immunotoxic or detrimental effects of the DC vaccination in exacerbating Graft-versus-host disease (GVHD). However, further clinical developments were hampered due to the complex, costly and inconsistent production of conventional DCs under good manufacturing practice (GMP) [12]. Production of conventional DCs requires several days of GMP culture, is difficult for large-scale production and faces difficulties towards good automated manufacturing practice (GAMP). Finally, the low viability and migratory properties of clinical-grade conventional DC after administration into patients is considered a major concern for their sub-optimal bio-distribution to LN and clinical potency [6].

During the past decade, lentiviral vectors (LV) have been intensively explored to enable persistent antigen expression in DCs to enhance immunization [13–15]. Integrase-defective lentiviral vectors (IDLV), which can potentially lower the genotoxicity risks mediated by viral insertional mutagenesis, are currently being tested experimentally as recombinant viral vaccines against infections, cancer and parasites [16–19]. We have previously shown in a series of studies that monocytes can be programmed with lentiviral vectors to self-differentiate autonomously into highly viable and activated DCs [20–23]. We have employed IDLV for co-expression of granulocyte-macrophage colony stimulating factor (GM-CSF), interferon (IFN)-α, and the immune dominant HCMV pp65 tegument antigen [20, 22]. This combination of transgenes enabled monocytes to autonomously become Self-differentiated myeloid-derived lentivirus-induced DC expressing pp65 (SmyleDCpp65) after a single overnight ex vivo gene transfer [20, 22]. Prime/boost immunizations with SmyleDCpp65 after transplantation of immune deficient NOD.Rag1^−/−^.IL2rγ^−/−^ (NRG) mice with human CD34^+^ stem cells resulted in remarkable acceleration of de novo immune reconstitution, LN regeneration, improved expansion of mature T and B cells and pp65-specific human T and antibody responses [22, 23].

Here, we pre-clinically showed in a simple and short fully GMP-compliant production scheme that SmyleDCpp65 cryopreservation and thawing were feasible and did not negatively affect characteristics and function of the cells. In addition, state-of-the-art analytical methods for QC and batch release, insertional mutagenesis assessment risk and potency characterization were established.

## Methods

### Cell lines

The HEK-293 (human embryonic kidney-293) cell line encoding the simian virus 40 (SV40) large T antigen (heretofore, 293T cells) was used for the production and characterization of lentiviral vectors (both research grade and GMP grade). A master cell bank (MCB) of 293T cells was established at EUFETS GmbH (Idar-Oberstein, Germany). Two randomly picked 293T MCB vials were tested for sterility, endotoxins, mycoplasma and adventitious viruses in full compliance with the GMP requirements of the local regulatory authorities. These safety tests have shown that the MCB was devoid of bovine, porcine and human viruses. HT1080 (human fibrosarcoma cell line) was used for titration of the lentivirus produced with GMP grade materials. 293T cells and HT1080 cells were cultured at 37°C and 5% CO_2_ in Dulbecco’s modified Eagle’s medium (DMEM, Invitrogen, Darmstadt, Germany) supplemented with 10% fetal bovine serum (FBS, HyClone, Fischer Scientific GmbH, Bonn, Germany). The reference K562 cells co-expressing HLA-A02^*^01 and pp65 were cultured in RPMI supplemented with 10% FBS, 1% penicillin/streptomycin and 1 mg/mL geneticin (Biochrom AG, Berlin, Germany).

### Lentiviral vectors and plasmids

ICLV-pp65 used for control experiments was produced as previously described [21]. IDLV-G2α2pp65 was produced by transient transfection of four plasmids containing the transfer plasmid RRL-CMV-G2α2pp65, the packaging plasmid pCDNA3.g/pD64V.4xCTE encoding the D64V mutation in the integrase gene, the packaging plasmid expressing Rev and the pMDG plasmids encoding for the vesicular stomatitis virus G glycoprotein (VSV-G) as described [20]. For GMP production of the virus, all plasmids were fully sequenced and produced by PlasmidFactory GmbH (Bielefeld, Germany) as ccc-supercoiled Grade plasmids (enzyme free and devoid of bovine derived material and certified for purity). IDLV-G2α2pp65 was produced under GMP conditions following standard operation procedure (SOP) established at EUFETS GmbH. On day 0, 40 stack cell factories were seeded with 293T cells and transfected with qPEI (Polyethylenimine) transfection reagent. 24 h after transfection, medium change and Benzonase treatment were performed. Supernatant containing virus was harvested 48 h after transfection (total volume 2,500 mL), filtered through 0.8 and 0.45 µm filters and purified by chromatography (CEX). Tangential flow filtration and dialysis was performed and the viral supernatant was concentrated approximately 33-fold (to 74 mL). The purified virus was filtered with 0.45 and 0.2 µm filters, the final product was aliquoted as 1 mL/vial and stored at −80°C.

### Titration of IDLV-G2α2pp65 by p24 analyses

Physical titers of the vectors produced (Research grade and GMP grade) were determined by quantifying the p24 HIV-I core protein by ELISA (QuickTiter™ HIV Lentivirus Quantitation Kit, BioCat, Heidelberg, Germany).

### Titration of the vector and analyses of vector copy numbers in monocytes by RT-q-PCR

For virus titration, HT1080 cells were transduced and genomic DNA was extracted from using the QiaAmp DNA blood mini kit (Qiagen, Hilden, Germany) according to the manufacturer’s instructions. For analyses of IDLV copy numbers in transduced monocytes, total DNA (tDNA) was extracted using the Epicenter Masterpure DNA isolation kit (Madison, WI, USA), with adaptations [24]. Cells lysates with incubated with proteinase K treatment, (45 min at 65°C), RNase A treatment (10 µg, 37°C for 30 min) and proteins removed by precipitation. The tDNA was precipitated from supernatants with isopropanol and solubilized in 85 µL of tris-buffer according to the manufacturer’s instructions. IDLV copy numbers were determined by real-time PCR as previously described [22, 25]. Shortly, 2 µL containing 100 ng of genomic DNA were added to 13 µL of RT-q-PCR mix [containing 7.5 µL of SYBRTaq mix with 1 µL of wPRE/PTB2 primer mix (wPRE forward: 5′-GAGGAGTTGTGGCCCGTTGT, wPRE reverse: 5′-TGACAGGTGGTGGCAATGCC or PTBP2 (polypyrimidine tract binding protein 2; PTBP2 forward: 5′-TCTCCATTCCCTATGTTCATGC, PTBP2 reverse: 5′-GTTCCCGCAGAATGGTGAGGTG) and 4.5 µL PCR grade, nuclease free water]. All samples were analyzed with StepOnePlus™ Real time PCR system (Applied Biosystems, Life Technologies, Darmstadt, Germany). The cycling conditions were 10 min at 95°C, 40 cycles of 15 s at 95°C, 20 s at 56°C and 30 s at 65°C. Results were quantified by making use of primer pair-specific real-time PCR efficiencies and by comparing sample CT values to a standard curve generated with the plasmid vector (pCR4-TOPO) containing the wPRE and PTB2 sequences. Data was analyzed by StepOnePlus™ software (Applied Biosystems).

### Research grade (RG) SmyleDCpp65 generation

Leukapheresis of non-mobilized HCMV-seropositive and HLA-A02*01 positive adult healthy donors was performed in accordance with study protocols approved by Hannover Medical School Ethics Review Board. Peripheral blood mononuclear cells (PBMNCs) were purified by sediment centrifugation (Ficol, Biochrome AG) and cryopreserved. CD14^+^ cells were selected using CD14 immunomagnetic microbeads (MACS, Miltenyi Biotec, Bergisch Gladbach, Germany) and processed as previously described [20, 22]. In short, CD14^+^ cells were pre-conditioned with hGM-CSF and hIL-4 (50 ng/mL each, CellGenix, Freiburg im Breisgau, Germany) for 8 h followed by transduction. IDLV-G2α (a bicistronic vector expressing GM-CSF and IFN-α, but no antigen) was used to generate “empty” SmyleDCs, while IDLV-G2α2pp65 (a tricistronic vector expressing GM-CSF, IFN-α and pp65) was used to generate SmyleDCpp65. The design and validation of these vectors were described previously [20, 22, 23]. For production of research grade SmyleDC or SmyleDCpp65, 2.5 μg/mL p24 equivalent of the respective vector were used to transduce 5 × 10^6^ monocytes at MOI of 5 in the presence of 5 μg/mL protamine sulfate (Valeant, Eschborn, Germany) for 16 h. After transduction, cells were washed twice with CellGro medium (CellGenix). Cells were cryopreserved in freezing medium containing 15.5% human albumin, 10% DMSO and 5%Glucose. Conventional IFN-α DCs were generated from monocytes by supplementing the medium with hGM-CSF and hIFN-α every 3 days.

### SmyleDCpp65 production with GMP compliant methods

Leukapheresis of non-mobilized HCMV sero-positive HLA-A02*01 and/or HLA-B07*02 healthy adult volunteers was performed with a COBE^®^ Spectra apheresis system. PBMCs were used fresh for selection of CD14^+^ monocytes with a GMP-compliant CliniMACS immunomagnetic separation system (Miltenyi Biotec). Quantitative and qualitative analyses of the selected CD14^+^ fraction and flow through were performed by flow cytometry. From the enriched CD14^+^ fraction, 1.5 × 10^8^ cells were resuspended in 25 mL of serum free CellGro DC medium (CellGenix) and seeded in a 100 mL bag (CellGenix). Cells were preconditioned with 25 mL of medium containing hGM-CSF and hIL-4 cytokines (50 ng/mL each, CellGenix) for 8 h. Cells were transduced with 7.5 × 10^8^ infective particles (multiplicity of infection of 5) in 50 mL medium containing Protamine sulphate (5 µg/mL). The bag was incubated at 37°C and 5% CO_2_ for 16 h. Next day, cells were washed three times with CellGro medium. After washing, cell number and viability was determined. Transduced cells were cryopreserved in aliquots of 2 × 10^6^ cell/mL/vial. Surplus, non-transduced monocytes were cryopreserved in aliquots of 2 × 10^6^ cell/mL/vial and 50 × 10^6^ cell/mL/vial and were used as controls for the characterization experiments. Sterility tests were performed with the “Bactec” system (BD Biosciences, Heidelberg, Germany). Three independent production batches were prepared with three independent leukaphereses obtained from three independent healthy adult volunteers.

### Analyses of thawed GMP grade SmyleDCpp65 by flow cytometry analyses

Three independent cryopreserved vials (2 × 10^6^ cell/mL/vial) of monocytes transduced with IDLV-G2α2pp65 in each production batch were analyzed directly at thaw (AT), or cultured in CellGro for 5 or 7 days at a concentration of 1 × 10^6^ cells/mL. Surface marker expression was analyzed by flow cytometry using the following monoclonal Ab conjugated with fluorochromes: Krome Orange-CD45 (clone J.33), APC750-CD14 (clone RMO52), PC7-CD11c (clone BU15), PE-CD86 (clone HA5.2B7), Pacific Blue-HLA-DR (clone Immu-357; all from Beckman Coulter, Krefeld, Germany), and APC-CD80 (clone 2D10; Miltenyi Biotec). Residual cell analyses were performed with the following Ab: APC700-CD3/CD19 (T and B cells; clones: UCHT1 and J3-119 respectively) and PC5.5-CD56 (NK cells; clone: N901 (HLDA6); all from Beckman Coulter). After staining and prior to acquisition, Flow-Count™ Fluorospheres (Beckman Coulter) were added to the cells to determine the absolute cell counts and to control the flow rate. Expression of pp65 was determined by intracellular staining and flow cytometry. Cells were stained with the following monoclonal antibodies Ab: Krome Orange-CD45 (clone J.33), Pacific Blue-CD14 (clone RMO52), PC7-CD11c (clone BU15), PE-CD86 (clone HA5.2B7), ECD-HLADR (clone Immu-357), and PC5.5-CD56 (clone N901 (HLDA6); all from Beckman Coulter). After surface staining, cells were washed and permeabilized with BD cytofix/cytoperm solution (BD Biosciences). After permeabilization, cells were incubated with FITC-conjugated mAB against HCMV-pp65 (clone: I1010D; Thermo Scientific, Germany) in a 1:20 dilution with BD perm/wash solution. Non-transduced monocytes are used as negative controls and conventional IFN-α DCs were used as positive controls. Acquisitions and analyses were performed by Navios™ Flow Cytometer and Navios™ analysis software (Beckman Coulter).

### Analyses of cytokines and transgene expression

SmyleDCpp65 produced under GMP-like conditions secreted several endogenous cytokines. We analyzed and quantified the levels of a set of commonly highly expressed cytokines in culture supernatants (GM-CSF, IFN-α, MCP1 and IL-8) by bead array luminex based kit according to the manufacturer’s protocol (Milliplex Milipore, MA, USA).

### Integration analyses by NGS

Integration analyses were performed by using LAM-PCR to identify the lentiviral vector-flanking genomic sequences as described [26]. Briefly, tDNA was extracted from the samples and two 50-cycle linear PCR amplification steps were carried out using biotinylated primers hybridizing to the 3-prime region of the long terminal repeats (LTR) of the vector. The biotinylated PCR-products were further captured with paramagnetic beads followed by second strand DNA synthesis, restriction digestion and ligation of a cohesive double-stranded linker sequence carrying a molecular barcode of 12 nt. Two nested PCR were then performed with linker and vector specific primers each complementary to one of the known ends of the target DNA. In 5′–3′ orientation, LAM-PCR products contained a LTR sequence, a flanking human genomic sequence and a linker cassette (LC) sequence. LAM-PCR amplicons were further prepared for MiSeq sequencing (Illumina, San Diego, CA, USA). Therefore, an additional PCR with special fusion-primers carrying MiSeq specific sequencing adaptors was performed. DNA barcoding was used to allow parallel sequencing of multiple samples in a single sequencing run. Libraries were mixed with an φX bacteriophage genome library to introduce diversity and optimize the sequencing run performance and sequenced using the Illumina MiSeq v2 Reagent Kit. The pair-end runs were initiated for Illunima’s sequencing by synthesis technology, including clustering, paired-end preparation, barcode sequencing and analysis. After completion of the run, base calling was performed on data, sequences were de-multiplexed and φX reads were filtered. Next generation sequencing (NGS) data processing dealt with the management of high-throughput data from Roche 454/Illumina MiSeq sequencing platforms and comprise two main goals: (1) data quality inspection and analysis, in which lentiviral vector sequences and other contaminants were trimmed; (2) integration site identification, in which all valid sequence reads are aligned to the genome of reference and valid ISs were retrieved.

### Characterization of SmyleDCpp65 potency after stimulation of T cells in vitro

The potency assays were based on activation of autologous T cells with SmyleDCpp65, which were produced with monocytes after CliniMACS selection. GMP grade-SmyleDCpp65 were compared with research grade (RG) SmyleDCpp65 and SmyleDC produced with monocytes from the same donor, but which were produced with IDLVs produced in the laboratory. Autologous CD3^+^ T cells from each of the three GMP-grade batches were isolated from the CD14^neg^ fraction (MACS positive selection, Miltenyi Biotec). CD3^+^ T cells were co-cultured with RG-SmyleDC, RG-SmyleDCpp65 or GMP-grade SmyleDCpp65 at a ratio of 10–1. Non-stimulated CD3^+^ T cells were used as negative control. CD3^+^ T cells stimulated for 16 h with 10 µg/mL PepTivator CMV-pp65 overlapping peptide pool (Miltenyi Biotec) were used as reference controls for the IFN-γ intracellular detection assay. Protein transport inhibitor cocktail (eBioscience, Frankfurt, Germany) was added to the cells 1 h after stimulation. After 16 h, T cells were harvested, stained with APC-conjugated anti-human CD3, Pacific Blue-conjugated anti-human CD4 and PECy7-conjugated anti-human CD8 antibodies. After fixation/permeabilization with Cyofix/perm (BD Biosciences) for 20 min at 4°C and washing, anti-human PE-IFNγ (eBioscience) was used for staining for 30 min. The cells were acquired by flow cytometry using LSRII (BD Biosciences) and analyzed by Flowjo^®^ software (Treestar Inc., Ashland, OR, USA).

### Statistical analysis

Parametric (t test) and non-parametric Mann–Whitney U test were used for determining statistical significance. All tests were two-sided, and *p* < 0.05 was considered significant. Data was analyzed with GraphPad Prism 5 software (GraphPad Software, Inc., CA, USA).

## Results and discussion

### Feasibility of IDLV-G2α2pp65 production under GMP-like compliant conditions

**Figure 1:**
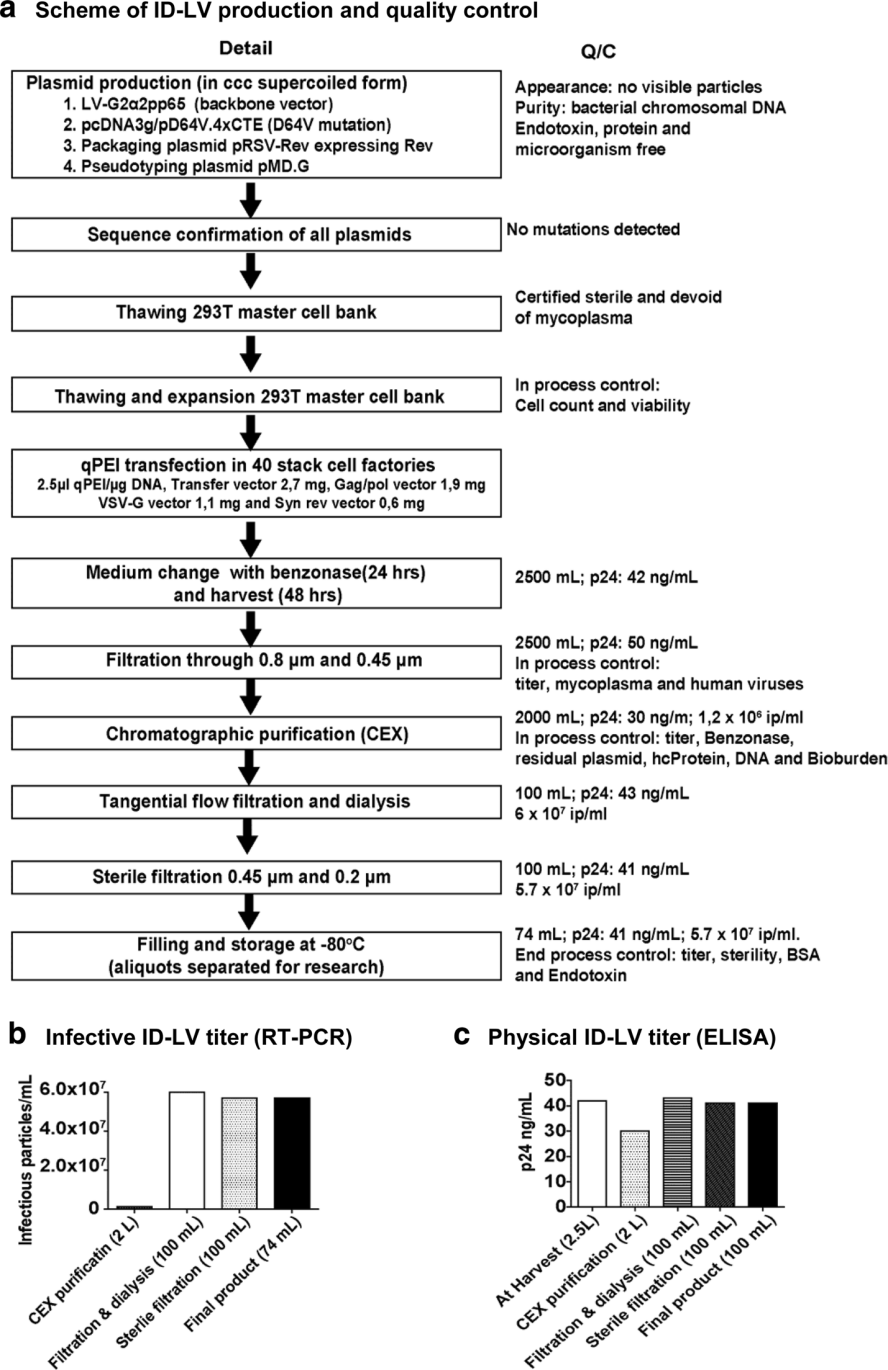
Standardized production of IDLV-G2α2pp65 under GMP compliant conditions up-scaling, recovery and titration. **a** Schematic representation of pilot batch of the lentiviral vector production performed under GMP compliant conditions. In process QC analyses are shown. **b** Infective titer of IDLV samples collected during different steps of GMP-compliant production. Indicator cells (HT1080) were transduced and the number of vector copies in genomic DNA was determined by RT-q-PCR. **c** Quantification of IDLV physical titer as p24 equivalent by ELISA.

We have previously demonstrated and validated a tricistronic vector IDLV-G2α2pp65 co-expressing simultaneously GM-CSF, IFN-α and pp65 (Additional file 1: Figure S1A) for generation of SmyleDCpp65 [23]. Advancing towards clinical grade SmyleDCpp65 for clinical trials, we evaluated the feasibility of vector production and cell generation under GMP compliant conditions. In collaboration with a contract manufacturing organization (CMO, EUFETS GmbH), we validated upstream and downstream processes and established standard-operating-protocols (SOPs) for production of the vector IDLV-G2α2pp65 by transfection of 293T cells. High purity ccc plasmids consisting the transfer vector (LV-G2α2pp65), the *gag/pol* vector harboring a D64V mutation (pcDNA3g/pD64V.4xCTE), the vector containing codon optimized REV (pRSV-REV), and the envelope vector expressing VSV-G (pMD.G) were used for transfection in 40 stack cell factories (Figure 1a). After transfection, viral supernatant was harvested and subjected to downstream purification (benzonase treatment, filtration, chromatographic purification, tangential flow filtration, sterile filtration, filling and storage). At each step of the purification process, a QC step was added to determine the recovery and yield of IDLV (Figure 1a). Infectious titer of IDLV-G2α2pp65 was determined by RT-q-PCR and the physical titer by quantifying the HIV-I core protein p24. Starting with 2 × 10^6^ infectious particles/mL (ip/mL; 2 L volume) after CEX purification, final product was concentrated by 33-fold in relation to the starting volume with a final titer of (from 2,500 to 74 mL; Figure 1a). The filtration and concentration steps did not alter the infective titer of IDLV. The final product showed a titer of 5.7 × 10^7^ ip/mL in a total of 74 mL (4.2 × 10^9^ infectious particles) (Figure 1b). On the other hand, physical p24 titer of IDLV-G2α2pp65 revealed major reduction after CEX purification, filtration and dialysis steps (Figure 1c), which was probably due to removal of empty particles and cell debris containing p24 during the purification process. Overall, GMP-grade IDLV-G2α2pp65 production and recovery demonstrated that IDLV production was not fundamentally different from ICLV production methods established by the same CMO.

### Feasibility of SmyleDCpp65 generation and cryopreservation under GMP-like compliant conditions

Since cryopreservation of SmyleDCpp65 could facilitate the production logistics, storage and performance of quality control analyses, we performed preliminary tests to evaluate the effects of cryopreservation immediately after IDLV transduction (representative example Additional file 1: Figure S1B-F). After thaw (AT), transduced cells were highly viable 7AAD^neg^ and pure CD14^+^ monocytes containing detectable IDLV copies. After culture for 7 days, SmyleDCpp65 maintained IDLV copies and the persistent gene transfer was associated with expression of the pp65 antigen. After culture, SmyleDCpp65 were still highly viable, down-regulated the expression of monocytic marker CD14^+^, up-regulated the expression of the DC marker CD11c and co-expressed the relevant molecules HLA-DR/CD86 and HLA-DR/CD80. Therefore, under good research practice, cryopreservation was not detrimental to recovery of self-differentiated SmyleDCpp65 after thawing.

**Figure 2:**
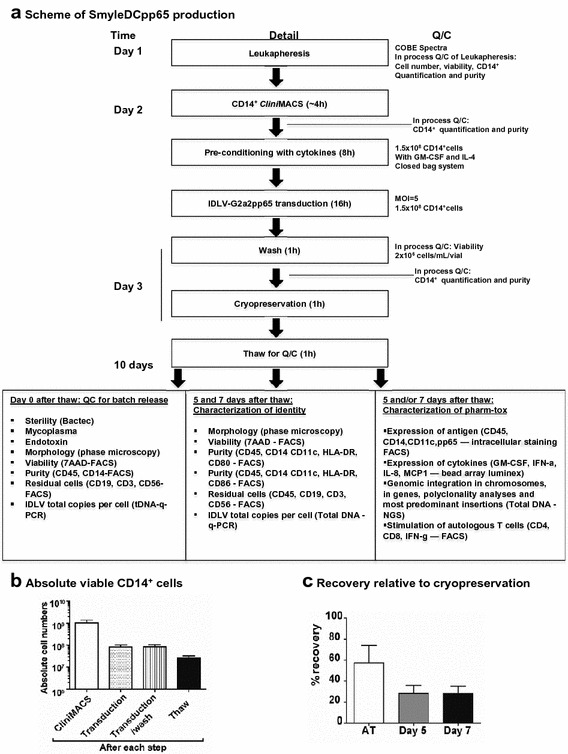
Standardized production of SmyleDCpp65 under GMP compliant conditions. **a** Schematic flow diagram representing the production of a pilot batch of SmyleDCpp65 generation under GMP-like conditions and analyses. CD14^+^ monocyte selections from three independent leukapheresis were performed by CliniMACS. 1.5 × 10^8^ monocytes were transduced in bags with 7.5 × 10^8^ infectious particles (MOI of 5). Transduced monocytes were washed and cryopreserved. Analyses on day 0 after thaw for QC and batch release and on days 5 and/or 7 for identity and pharm-tox are shown. **b** CD14^+^ cell recovery as viable cell numbers after each step of production process and AT (extrapolated). **c** Percentage recovery of viable cells AT, days 5 and 7 after culture in medium without exogenous addition of cytokines (3 independent runs; n = 3 for each run).

Therefore, we proceeded with upscaling the production of cryopreserved SmyleDCpp65 with SOPs using the vector generated under GMP-like conditions. The standardized production was validated in three independent runs. Ex vivo monocyte manipulation starting with leukapheresis at the clinical center and proceeding with transportation under controlled conditions to the CMO for CD14^+^ isolation, cytokine preconditioning, transduction, wash and cryopreservation could be fully achieved in only 3 days (Figure 2a). In process QC steps were included after each step in the production process to assess the cell viability and recovery (Figure 2a). In order to assess the purity, viability and recovery of monocytes from the leukapheresis until thaw, in process QC steps were added after each step in the production process. Three independent CD14^+^ CliniMACS^®^ enrichments were performed with leukapheresis obtained from donors, resulting in >80% viable CD14^+^ purity (Table 1). A range of 0.47–1.00 × 10^9^ CD14^+^ cells were recovered from 1.47–2.29 × 10^9^ PBMNCs (Figure 2b). 1.5 × 10^8^ CD14^+^ monocytes were used in transductions with GMP-grade IDLV-G2α2pp65 at an MOI of 5 (7.5 × 10^8^ infectious particles). After transduction and washing, 0.60–1.23 × 10^8^ viable monocytes were recovered (Figure 2b). SmyleDCpp65 were cryopreserved as 2 × 10^6^ cells in 1 mL per vial as the final cell product. A fraction of the CD14^+^ cells that were not transduced were equally cryopreserved at 2 × 10^6^ cells per mL per vial and were used as negative controls for the QC analyses. Sterility tests were negative for bacterial and fungal contamination (data not shown). After cryopreservation, samples were transported to our clinical center under controlled conditions for further QC and characterization analyses. At independent time points, three vials from each of the three SmyleDCpp65 production batches were thawed and characterized for sterility, viability, identity and purity. At thaw (AT), a range of 0.60–1.73 × 10^6^ cells corresponding to 30.0–86.5% of the cryopreserved cells were recovered (Figure 2c). Cell counts performed at the CMO demonstrated that 30% of the cells seeded AT maintained viable after ex vivo culture for 5–7 days (Figure 2c).

**Table 1:** Range of cell recovery at different steps of processing and after thawing (n = 3)

Donor	# PBMNCs in leukapheresis	# CD14^+^ selected (% of total leukapheresis)	# CD14^+^ transduced (% from selected CD14)	# Cells after transduction (% recovery from input)	# Cells cryo-preserved/mL/vial	# Cells after thawing (day 0) (% from cryopreserved)	Transduction efficiency LV copies/cell	% pp65 expression (day 7 CD45^+^/CD11c^+^)	% Activated DCs− (day 7 CD45^+^/CD11c^+^/CD80^+^/HLA-DR^+^)
#1	6.08 × 10^9^	1.01 × 10^9^ (17%)	1.5 × 10^8^ (15%)	6.90 × 10^7^ (46%)	2 × 10^6^	0.6 × 10^6^ (30%; n = 2)	n.a.	44.40	93.50
#2	2.07 × 10^10^	1.62 × 10^9^ (8%)	1.5 × 10^8^ (9%)	5.95 × 10^7^ (40%)	2 × 10^6^	1.16 × 10^6^ (58%; n = 3)	0.66	39.40	95.50
#3	9.07 × 10^9^	4.67 × 10^8^ (5%)	1.5 × 10^8^ (32%)	1.23 × 10^8^ (82%)	2 × 10^6^	1.73 × 10^6^ (87%; n = 3)	1.31	44.50	97.20
Mean ± SEM	1.19 × 10^10^ ± 4.46 × 10^9^	1.03 × 10^9^ ± 3.3 × 10^8^ (10%)	1.5 × 10^8^ (19%)	8.4 × 10^7^ ± 1.9 × 10^7^ (56%)	2 × 10^6^	1.16 × 10^6^ ± 3.3 × 10^5^ (58%)	0.99	42.8 ± 1.7	95.4 ± 1.1

Standardized developmental runs of SmyleDCpp65 production were performed as triplicate independent runs using monocytes from three different healthy adult volunteers. Cell numbers and percent recovery after each production step are shown. Transduction efficiency and variability in the DC immune phenotype are shown.

**Figure 3:**
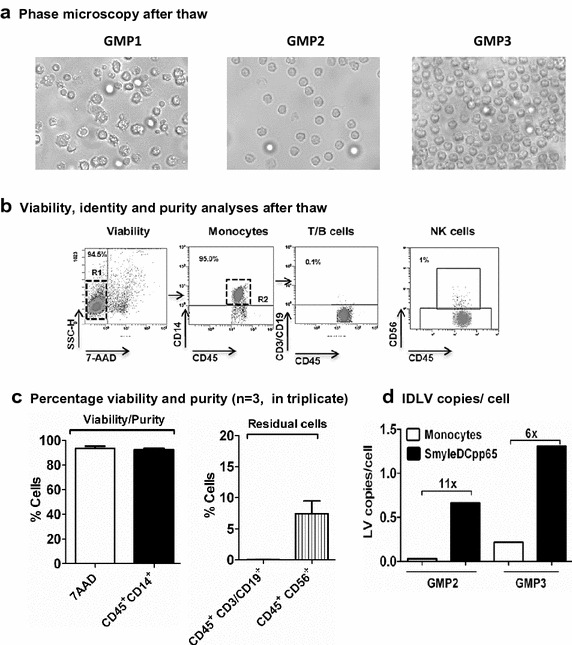
Characterization of transduced CD14^+^ monocytes after thaw (AT). **a** Phase microscopy of transduced CD14^+^ cells AT showing viable monocytes. **b** Representative example of flow cytometry gating for QC and batch release criteria, showing high viability, purity and expected monocyte characteristics of the product. **c** Overall viability (7AAD^neg^), purity (CD45^+^CD14^+^) and quantification of residual cells (T cells and B cells: CD45^+^ CD3^+^/CD19^+^ and NK cells: CD45^+^CD56^+^) for the three SmyleDCpp65 production runs (GMP1: n = 2; GMP2: n = 3; GMP3: n = 3 independent analyses). **d** Detection of IDLV copies in tDNA extracted from transduced SmyleDCpp65 and cognate non-transduced monocytes (both groups AT) by RT-q-PCR. PCR signal in transduced monocytes compared with baseline is indicated. *Bar graphs* indicate mean and the *error bars* indicate mean ± SEM.

The most relevant criteria to ensure quality of the cell product after filling, cryopreservation and thawing are purity and viability. Thus, standardized quality control analyses to define these parameters were performed in collaboration with a GMP development unit in our institution. After thawing, SmyleDCpp65 showed undifferentiated monocyte morphology and high viability (>90% 7AAD), expressed monocyte markers (>90% CD45^+^ and CD14^+^) and showed low frequency of residual T and B cells (<1% CD45^+^/CD19^+^/CD3^+^) (Figure 3a–c). Unexpectedly, as we intended to evaluate the frequency of residual natural killer cells with the CD56 marker, we observed a consistent population (8%) of CD56^+^ cells, which may relate to the recently reported CD56^+^ IFN-α-induced dendritic cells shown to efficiently stimulate autologous Vγ9γδT cells [27].

As the biological activity of SmyleDCpp65 depends on the successful transduction, detection of IDLV copies is a primary criterion for QC of the process. For the first batch of transduced monocytes (“GMP1”), there was a technical problem with the liquid nitrogen storage of samples at the CMO site. Thus, some of the samples of GMP1 were lost and could not be further analyzed regarding IDLV integration. AT, tDNA was isolated for GMP2 and GMP3 batches. Non-transduced monocytes obtained from the same donors were used as baseline control. IDLV copies could be detected in the GMP batch 2 (0.66 copies per cell, 11× above assay baseline) and in the GMP batch 3 (1.31 copies per cell, 6× above assay baseline) (Figure 3d). All together, these results demonstrated a robust and consistent 3-day manufacturing method for generation of cryopreserved SmyleDCpp65 under GMP.

### Characterization of SmyleDCpp65 after in vitro culture

**Figure 4:**
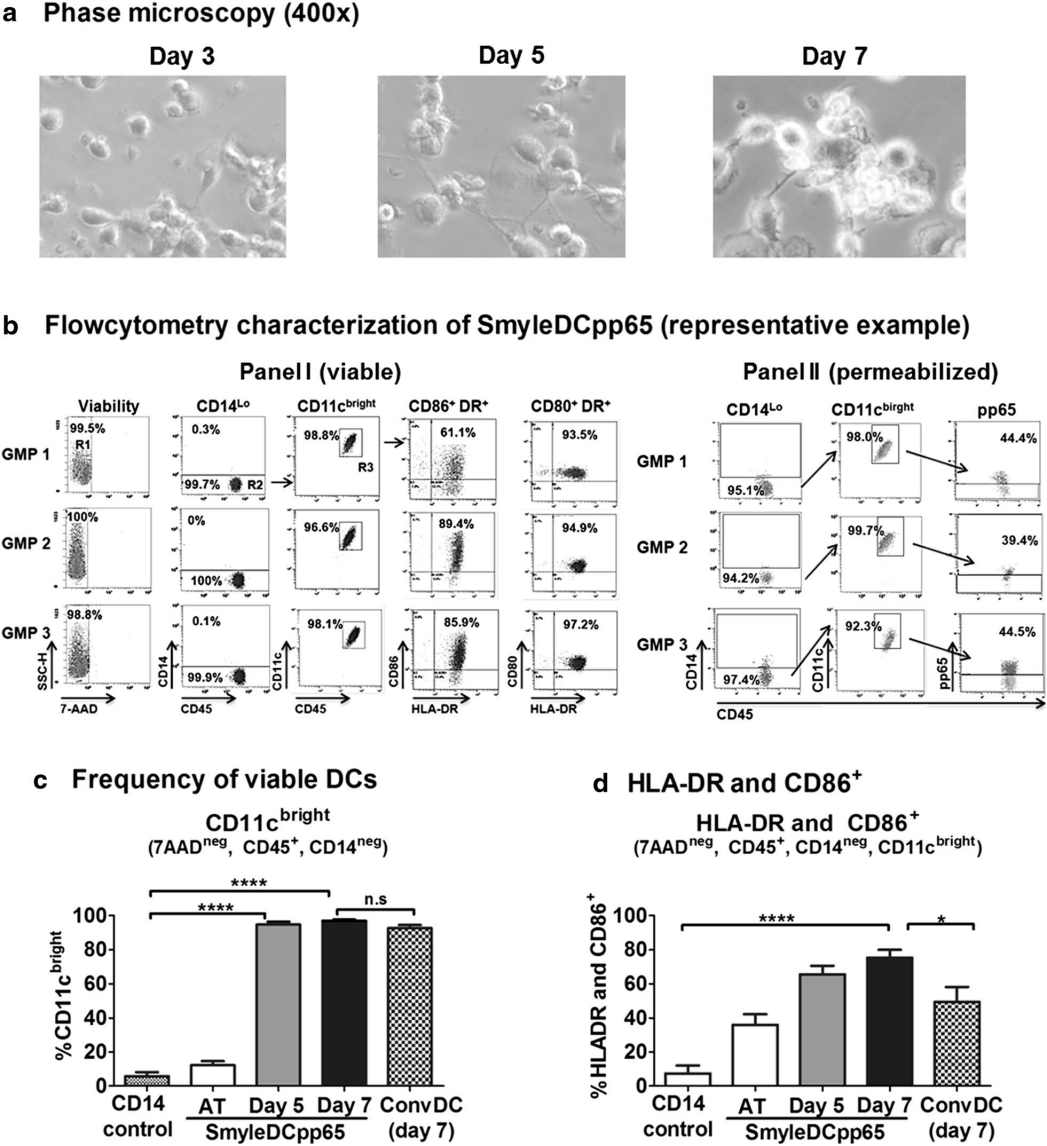
Characterization of SmyleDCpp65 after in vitro culture for 5 or 7 days without exogenous cytokines. **a** Morphology analyses by phase microscopy. SmyleDCpp65 thawed and cultured for 3, 5 or 7 days showing high viability throughout and acquisition of typical DC morphology and cell clustering from day 5 onwards. **b** Methods for flow cytometry analyses for characterization of SmyleDCpp65 identity [*Panel I* (viable)—DC phenotype] and potency [*Panel II* (permeabilized)—pp65 expression] and representative examples for each GMP batch analyzed. **c** Average frequency of viable DCs (CD14^−^, 7AAD^−^, CD45^+^ CD11c^bright^ cells) for all analyses of GMP batches combined. **d** Average frequency of activated DCs co-expressing HLA-DR/CD86. Data represent the combined data obtained for three independent SmyleDCpp65 production runs, two independent analyses for GMP1 and three independent analyses for GMP2 and GMP3 (in total 8 analyses). *Bar graphs* indicate mean and *error bars* indicate mean ± SEM. *p < 0.05, **p < 0.01, ****p < 0.0001.

**Figure 5:**
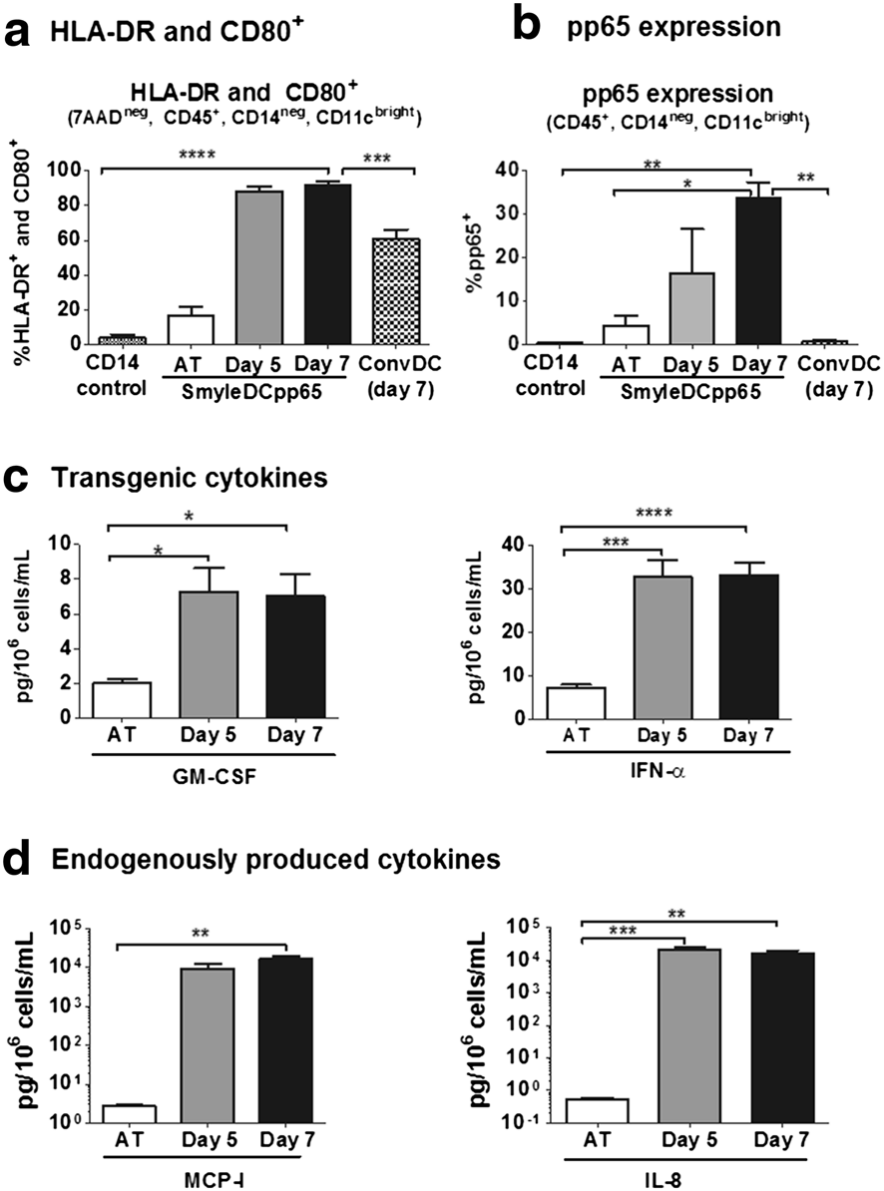
Characterization of SmyleDCpp65 after in vitro culture for 5 or 7 days without exogenous cytokines. **a** Average frequency of activated DCs co-expressing HLA-DR/CD80. **b** Average frequency of pp65^+^ cells detected after intracellular staining and FACS. **c** Concentration of transgenic cytokines GM-CSF and IFN-α in cell supernatants harvested AT, and on days 5 and 7 after thaw (pg/10^6^ cells/mL). **d** Concentration of endogenously up-regulated MCP-1 and IL-8 in cell supernatants harvested AT, and on days 5 and 7 after thaw (pg/10^6^ cells/mL). Data represent the combined data obtained for three independent SmyleDCpp65 production runs, two independent analyses for GMP1 and three independent analyses for GMP2 and GMP3 (in total 8 analyses). *Bar graphs* indicate mean and *error bars* indicate mean ± SEM. *p < 0.05, **p < 0.01, ****p < 0.0001.

Analytical validation methods were established to demonstrate parameters of the cells after thawing and in vitro culture such as morphology, high viability, self-differentiation into an activated DC phenotype, expression of pp65 and cytokines. Cells were thawed, seeded at 10^6^ cells/mL, and re-analyzed immediately after thaw (day 0) or after 5 and 7 days of culture. Three independent cryopreserved SmyleDCpp65 vials from each production batch were thawed and analyzed and corresponding triplicates of freshly thawed monocytes and conventional monocyte-derived DCs from the same donor were used as control parameters for the flow cytometry analyses. While non-transduced monocytes showed clear viability loss after 3 days of culture in absence of added cytokines, SmyleDCpp65 maintained viable and progressively showed morphologic changes towards DC differentiation from days 3–7 of culture (Figure 4a). Two panels of antibodies were developed for flow cytometry characterization of the activation status of SmyleDCpp65 (Panel I; viable), and to detect the expression of the pp65 antigen in DCs (Panel II; permeabilized) (Figure 4b; Table 2). For the activated DC panel, CD45^+^ hematopoietic cells were stained for viability (7AAD), the monocyte marker (CD14, down-regulated) and DC markers (CD11c, up-regulated). Activated SmyleDCpp65 were identified as 7AAD^neg^, CD45^+^, CD14^neg^, CD11c^high^, HLA-DR^+^, CD86^+^ or 7AAD^neg^, CD45^+^, CD14^neg^, CD11c^high^, HLA-DR^+^, CD80^+^ (Figures 4c, d and 5a). Hence, SmyleDCpp65 cultured for 5 and 7 days, down-regulated CD14 expression (AT: >95%; day 5: 4%; day 7: <1%), expressed high levels of DC markers CD11c (12.2% AT; day 5: 94%; day 7: 97%; Figure 4c), co-expressed HLA-DR/CD86 (36% AT; day 5: 65.58%; day 7: 75.45%; Figure 4d) and highly expressed HLA-DR/CD80 (16.6% AT; day 5: 88.25%; day 7: 91.71%; Figure 5a). Although both CD86 (B7-2) and CD80 (B7-1) are key co-stimulatory molecules both providing signaling to T cells through binding to the co-stimulatory receptor CD28 or the co-inhibitory receptor CTLA-4, expression of CD86 is stable and can be found in immature DCs, whereas CD80 expression is upregulated and a hallmark of active DCs [28]. Residual cells were identified by gating on viable cells (7AAD^neg^) and stained for CD3, CD19 and CD56. For qualitative analyses of pp65 expression in DCs, non-transduced monocytes were used as for setting up the negative controls gates, and freshly thawed K562/pp65 were used as positive controls for pp65 expression (Additional file 2: Figure S2). Cells were stained with surface markers, permeabilized and stained for pp65. Frequency of pp65^+^ expressing DCs was represented as CD45^+^, CD14^neg^, CD11c^high^ and pp65^+^ (AT: 4.28%; day 5: 16.4%; day 7: 33.71%; Figure 5b). Thus, expression of pp65 continuously increased during culture. Bead array analyses of SmyleDCpp65 cell culture supernatants demonstrated only baseline levels of secreted transgenic cytokines AT, whereas detectable levels of GM-CSF (day 5: 7.26 and day 7: 7.02 pg/10^6^ cells/mL) and IFN-α (day 5: 32.93 and day 7: 33.25 pg/10^6^ cells/mL) could be measured in the cell supernatant only after 5 or 7 days of culture (Figure 5c). In addition, high levels (>10^4^ pg/10^6^ cells/mL) of endogenous up-regulated monocyte chemotactic protein-I (MCP-I) and IL-8 were detectable in cell culture supernatants harvested on days 5 and 7 (Figure 5d). MCP-I and IL-8 are chemokines and we have previously demonstrated dramatic up-regulation of these cytokines in lentivirus-induced DCs [22, 29]. Therefore, analyses of quality control by these flow cytometry and luminex

systems provided a consistent pattern of the product and confirmed identity and immunologic potency of

SmyleDCpp65.

**Table 2:** Panels for flow cytometry characterization of SmyleDCpp65

	Viability	Pan maker	Dendritic cell	Activated dendritic cell	Cell impurities	Intracellular detection of pp65 antigen
Panel I	SSC & 7AAD^–^	CD45 (KromeOrange)	CD14 (APC 750) CD11c bright (PC7)	HLA-DR (Pacific Blue)/CD86 (PE) HLA-DR (Pacific Blue)/CD80 (APC)	CD3 & CD19 (APC700)	n.a.
Panel II	n.a.	CD45 (KromeOrange)	CD14 (APC750) CD11c bright (PC7)	n.a.	CD56 (PC5.5)	Anti-pp65 Ab plus secondary Ab (FITC)

Markers used in the characterization of SmyleDCpp65 after thawing and in vitro culture.

*Panel I* markers and fluorochromes used for detection of surface molecules in viable, non-permeabilized cells, *Panel II* markers and fluorochromes used for detection of surface molecules and pp65 in permeabilized cells.

### Analyses of IDLV copies and genomic integrations in SmyleDCpp65 and polyclonality

**Figure 6:**
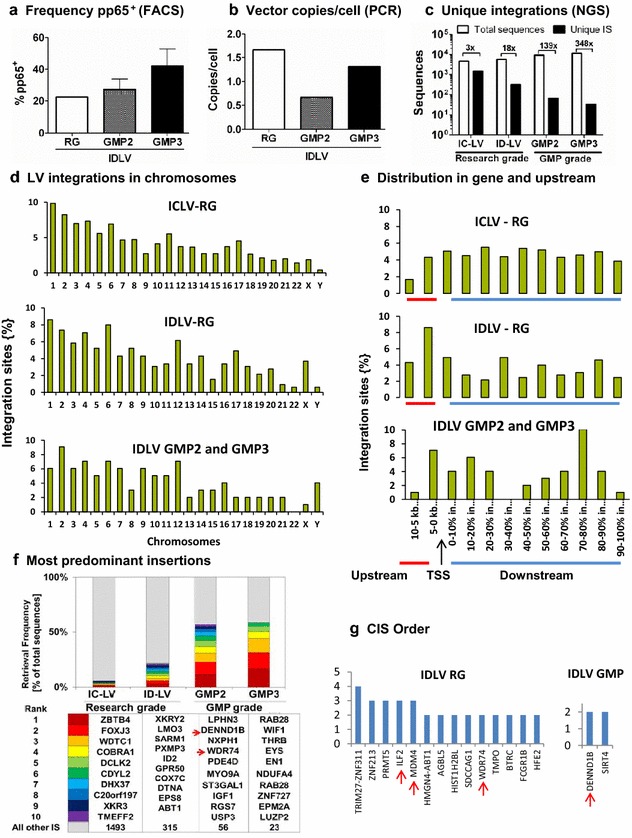
Analyses of IDLV genomic integrations in SmyleDCpp65. Cells from different production batches (RG-ICLV, RG-IDLV, GMP2 and GMP3) were thawed and cultured for 7 days without exogenous addition of cytokines. Total DNA (tDNA) was extracted from the cells for q-PCR or for LAM-PCR followed by high throughput integration analyses of LV sequences. **a** Reference analyses of pp65^+^ cells detected by FACS for each cell product. **b** Analyses of number of vector copies/cell performed by q-PCR (amplification of WPRE sequences). **c** Total matched sequences and unique integration sites (IS) analyses performed after LAM-PCR (LV sequences amplified with LTR primers) of the tDNA followed by NGS. Fold reduction from the total matched sequences to IS is indicated. **d** Frequency of IDLV integrations per chromosome, showing correlation with chromosome size. **e** Distribution of IDLV integrations upstream of transcription start sites (TSS *arrow*) or within genes. **f** Ten most pre-dominant clones detected in SmyleDCpp65 (RG-ICLV, RG-IDLV and GMP batches 2 and 3). *Colored columns* represent retrieval frequency as percentage of total sequences. *Larger colored bars* represent higher frequencies of integration sites clustering in the proximity of that gene. *Lower panel* Ranking of the 10 most pre-dominant clones with their corresponding color code and gene ID. *Red arrows* indicate frequent insertion sites observed in DENND1B and WDR74 for GMP2. **g** Common Integration Site (CIS) statistical analyses performed for RG-IDLV and GMP2 and GMP3 batches combined. *Red arrows* indicate recurrent insertion sites observed in the analyses (LIF2, MDM4, WDR74, DENND1B).

Although IDLVs are currently explored in experimental animal models to be used as recombinant viral vaccines against infections, cancer and parasites [16–18], there is still a lack of studies in human preclinical studies correlating transgene expression, vector copy numbers and pattern of residual integration. Due to the potential risk of insertional mutagenesis, this is a serious preclinical requirement to be observed. Residual lentiviral integration of IDLV was characterized in hepatocytes of mice infused with IDLV, demonstrating that the risk was greatly reduced but not totally eliminated [30]. At day 7 after culture, we could estimate based on detection of pp65 antigen as a qualitative method, that at least 30–40% of the cells produced under GMP were transduced (Figure 6a). Analyses of different batches of cells by quantitative RT-PCR demonstrated that IDLV copy content per cell ranged between 0.5 and 1.2 copies per cell (Figure 6b). We subsequently compared the LV integration pattern observed in day 7 SmyleDCpp65 transduced with vectors produced as research grade (ICLV versus IDLV) and as GMP grade (GMP-2 and GMP-3). DNA was extracted and analyzed by LAM-PCR and NGS and the clonal frequencies of the transduced monocytes maintained in culture for 7 days was monitored with a high throughput IS analysis [26]. The number of total vector sequences that could be retrieved for the four groups were in similar orders of magnitude (0.5–1.0 × 10^4^ sequences, Figure 6c). However, the detectable number of unique insertion sequences (IS) was much higher for RG-ICLV (>1,000) than RG-IDLV (335). Unique IS detectable for GMP2-IDLV and GMP3-IDLV combined were even lower, in total 99 sequences (Figure 6d). This indicated that only residual genomic lentiviral integration sites could be observed after IDLV gene transfer of monocytes under GMP-compliant conditions. Whereas for the research-grade vectors IDLV integrations were found in all chromosomes and frequencies were roughly correlated with chromosome size, interestingly no integrations were found in chromosome 22 for the two GMP-grade batches of SmyleDCpp65 (Figure 6d). As expected, ICLV integrated sequences were found in higher frequency in genes than upstream of the transcription start site (TSS) (Figure 6e). Surprisingly, for the RG IDLV sample, vector integrations were most frequent up to 5 kb upstream of the TTS. In turn, integrations detectable in the two GMP SmyleDCpp65 batches show a random distribution, but mostly in genes (Figure 6e). Although one could speculate that IDLV pattern of integration may indeed be more random than ICLV in monocytes, we would have to compare more samples for further analyses. Nevertheless, a relevant information was that IDLV genome integrations in gene loci were random and highly diverse among all RG and GMP samples analyzed. The research grade vectors showed higher polyclonality, but this may be due to the fact that there were more sequences to be analyzed. The 10 most frequent identified gene loci among the four samples were discordant and did not occur near oncogenes (Figure 6f). Tracking of the most common clones based on a Common Integration Site (CIS) statistical models [31–33] showed that, for RG-IDLV transduction, only one integration could be observed as 4th order (4 integrations within 100 kb genomic region, one of them in TRIM27), four integrations as 3rd order (3 integrations within 50 kb, with integration in WDR74 among others) and 9 integrations as 2nd order (2 integrations within 30 kb, with integration in DENND1B). Overall, these analyses confirmed previous observations that LV integrations are random showing a non-biased integration profile not matching a known biased integration sequences assigned for gamma-retroviral genotoxicity such as LMO2 [34]. Long-term cultures (30 days) of research-grade SmyleDCpp65 (produced with IDLV or ICLV without the cryopreservation step) were longitudinally harvested on days 10, 20 and 30, showing progressive reductions of LV copies (Additional File 3: Figure S3A). Pooling these time-points for each vector type, we observed that most of the ICLV and IDLV integrations combined were in gene ±10 kb outside genes (Additional File 3: Figure S3B-D). Analyses of the cultures maintained for up to 30 days showed also a consistent random integration in genes and polyclonality (Additional File 3: Figure S3C-D). Integration sites that showed up more than once in these analyses were WD Repeat-Containing Protein 74 (WDR74), ZNF37A, SEL1L, ILF2, but these genes are not known as proto-oncogenic. Notwithstanding, a common insertion of IDLV in the analyses of GMP batches were observed in WDR74 genes (Figure 6f, g), but insertional mutagenesis involved with the human locus was not described (NCBI Gene ID: 54663, updated on 17-Mar-2015). Furthermore, since monocytes and DCs are mostly post-mitotic and non-replicating cells, and it is unlikely that a biased integration pattern could predispose to insertional mutagenesis and genotoxic effects.

### Stimulation of autologous T cells with SmyleDCpp65 in vitro

**Figure 7:**
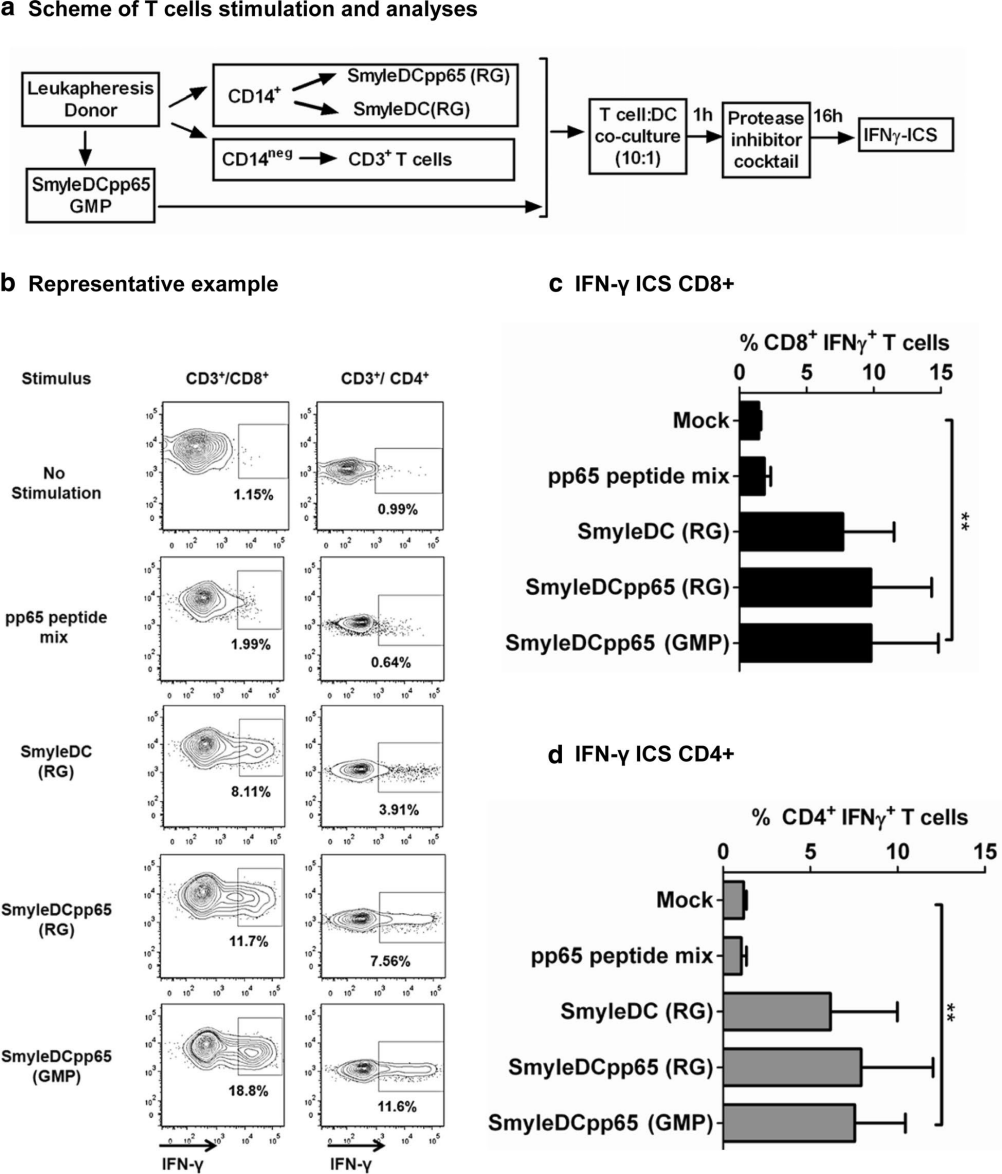
Characterization of SmyleDCpp65 potency by T cell stimulation in vitro. A 16 h IFN-γ catch assay based on flow cytometry analysis was used to evaluate whether SmyleDCpp65 produced under GMP-like compliant conditions (harvested on day 7 after thawing) could activate autologous CD3^+^ T cells (n = 3). No antigen (no stimulation) and stimulation with a pp65 peptide mix were used as controls. **a** Schematic representation of T cell stimulation assays performed with SmyleDC or SmyleDCpp65 (research grade, RG) or with cognate SmyleDCpp65 (GMP1, GMP2, GMP3). **b** Representative example showing gating and analyses of CD8^+^ and CD4^+^ T cells producing IFN-γ after different stimulations. **c** *Bar graph* showing the frequencies of CD8^+^ T cells and **d** CD4^+^ T cells producing IFN-γ. Results are indicative of three independent experiments. *Bars* indicate mean and *error bars* indicate mean ± SEM. **p < 0.05.

Ultimately, in order to demonstrate that SmyleDCpp65 produced under GMP-compliant conditions were functional in stimulating T cells, SmyleDCpp65 produced from the three donors were thawed, cultured for 7 days and used to stimulate autologous selected CD3^+^ T cells (Figure 7a). The donors used for these studies were all HCMV sero-positive and thus contained memory T cells reactive against pp65 epitopes. Non-stimulated T cells and cells pulsed with a pp65 peptide mix were included as experimental parameters. RG SmyleDC and SmyleDCpp65 produced with CD14^+^ cells from the same donor were used as positive controls. 16 h after stimulation, intracellular staining of IFN-γ and flow cytometry analyses were performed (Figure 7b, representative example). T cells stimulated with SmyleDCpp65 produced as GMP resulted in significant increases in frequencies of IFN-γ producing CD8^+^ T cells (SmyleDCpp65: 9.8%; versus unstimulated, *p* < 0.05) and CD4^+^ T cells (SmyleDCpp65: 7.5%; versus unstimulated *p* < 0.05) (Figure 7d). The T cell stimulation was modestly higher for SmyleDCpp65 (GMP or RG) than for SmyleDC (which can still activate T cells homeostatically, but lack the antigen). Under these assay conditions, the pp65 peptide mix loading on the T cells did not stimulate IFN-γ production in CD4^+^ or CD8^+^ T cells above the non-stimulated T cell control group. Therefore, these assays confirmed the functionality of GMP-grade SmyleDCpp65 to stimulate T cells in vitro.

## Conclusion

For future clinical developments of DCs to be used in conjunction with HSCT, novel enabling technologies for more potent and viable cell products, easy production and cryopreservation and well-defined specifications for quality control are needed. In order to fulfill these pre-requisites, here we advanced towards clinical development of SmyleDCpp65 under GMP-compliant conditions. SmyleDCpp65 can be genetically induced ex vivo, self-differentiate in vivo, are longer lived and produce combined antigenic effects for immune stimulation (cytokines, antigen, donor-matched MHC). GM-CSF and IFN-α are cytokines broadly used as generic drugs and biologic response modifiers against leukemia, and their safety and pharmacology have been extensively characterized. The pp65 HCMV antigen was evaluated in different types of vaccines in clinical trials and demonstrated potent T cell stimulatory properties not associated with immune toxicity [5]. In addition, pp65 is abundantly expressed early and late after HCMV infection, making it an appropriate antigenic target for CTLs. Proof-of-concept pharm-tox studies of SmyleDCpp65 in humanized mouse models of HSCT demonstrated accelerated immune reconstitution along with immunological potency and safety [22, 23]. In follow-up to these previous in vivo findings, the most important features of this current work are:

1. The feasibility to produce IDLV under GMP-compliant conditions: Our methods described here delineated the clinical grade up-scaling, standardized production, cryopreservation and QC for future clinical trials. Up-scaling the production of the IDLV and cell transduction were readily reproducible when performed by a CMO at GMP level. Our results demonstrated that production of IDLV with GMP-compatible methods was quite comparable to what has been reported for production of ICLV under GMP [35, 36]. The downstream processing of the vector did not alter the infectivity or the biological activity of the IDLV to reprogram monocytes. Currently, the most limiting factor for generation of the SmyleDCpp65 is the high costs of IDLV produced under GMP. New technologies driven by market competition (such as packaging cell lines and optimized up and down-stream processes) can potentially reduce the price of lentiviral production.
2. Use of fresh leukapheresis samples for SmyleDCpp65 manufacturing under GMP-compliant conditions and subsequent cryopreservation: With a standardized and simplified 3-day SmyleDCpp65 production method, approximately half of the transduced monocytes were recovered after 28 h of ex vivo manipulation. For three independent runs, we recovered 40, 46 and 82% of the monocytes used for transduction, which for developmental runs is a satisfactory result (Table 1). Thawing of the cells resulted in 30, 58 and 87% of viable cells, indicating that the cell handling procedures were technically continuously improved during these three feasibility runs (Table 1). Our protocol was established using 1.5 × 10^8^ monocytes. After transduction, freezing and thawing, we recovered approximately 8 × 10^7^ viable cells. This is a realistic number for QC testing and use in patients (the target clinical dose will be 1 × 10^6^ viable cells for immunization). Non-mobilized donor-derived monocytes are not the limiting factor for SmyleDCpp65 production, since after leukapheresis, we can select approximately 1 × 10^9^ monocytes. Incidentally, SmyleDCpp65 could also be produced with a fraction (10–15%) of the mobilized stem cell apheresis, such that only one apheresis would be needed. This way, the short time needed to complete production and quality control of SmyleDCpp65 would facilitate this cell therapy to be available for administration shortly after HSCT.
3. Detailed quality control for identity and characterization: For preclinical quality assurance, we developed the specification of the parameters for QC of cryopreserved/thawed SmyleDCpp65. Inter and intra-experimental variations detected for the three pilot SmyleDCpp65 lots after thaw were small regarding purity of the monocytes recovered. Non-clinical characterization of cultured SmyleDCpp65 using multicolor immunophenotypic panels (Table 2) showed remarkable reproducibility for viability and identity parameters on day 7 after in vitro culture (Table 1). An in vitro 16 h co-culture system followed by an IFN-γ catch assay measured by flow cytometry showed potency of GMP-grade SmyleDCpp65 to stimulate autologous CD4^+^ and CD8^+^ T cells.
4. Detailed analysis of the lentiviral vector integration: LVs have been shown to be safe in many gene therapy clinical trials for gene replacement in HSC [35–39] and have been vastly explored for transduction of mouse and human DCs in vitro [15]. A concern for LV-based gene transfer is insertional mutagenesis in progenitor myeloid cells. It was shown previously that HIV DNA integration in macrophages was favored in active transcription units [40]. LV integration profiles differed between human and rodent post-mitotic tissues [41], and therefore these analyses cannot be generalized, but analyzed on a case-by-case manner. SmyleDCpp65 are post-mitotic cells and do not replicate, enabling IDLV copies to be maintained also as episomal or residual integrated copies. Analyses of residual IDLV integration sites in GMP-grade SmyleDCpp65 showed a non-modal distribution upstream and downstream of transcription start sites. Surprisingly, research-grade SmyleDCpp65 generated with IDLV showed a more pronounced presence of vector integrations in a region of 5 kb upstream of transcription start site. We hypothesize that the purified IDLV preparation (i.e. free from empty viral particles containing p24) favored a random integration pattern, whereas non-purified particles could potentially tether to open chromatin at promoter/enhancer regions. For subsequent studies with a larger vector lot, we plan to assess the risk of generating replication competent lentivirus (RCL) as this is a required regulatory step for QC [42].

In summary, these results opens perspectives for the broad usage of this individualized cell vaccine therapy towards clinical trials to improve immune reconstitution and protection against HCMV after HSCT. This will be a first-in-man evaluation of an advanced therapy-medicinal product (ATMP) in an interventional, multicenter, prospective, randomized, open label, dose-escalating study assessing the safety, maximum tolerated dose and feasibility. The Phase I trial will include patients with AML, myelodysplastic syndrome or multiple myeloma transplant-recipients in remission at high risk of HCMV reactivation [seropositive recipients (R+) receiving HLA-matched stem cells from seronegative donors (D−)]. The primary objective will be to test the hypothesis that SmyleDCpp65 immunizations are safe, i.e. will not lead to an increase in incidence of acute GVHD, death, infections or occurrence of RCL. The secondary objective will be to test the hypothesis that SmyleDCpp65 immunizations will stimulate anti-HCMV immune responses and earlier quantitative and qualitative T and B cell reconstitution after transplantation.

## Additional files

Additional file 1: 
**Figure S1.** Feasibility of cryopreservation. (A) Tricistronic IDLV encoding for hGM-CSF, hIFN-α and CMV-pp65 protein used to generate SmyleDCpp65. (B) Scheme of SmyleDCpp65 generation. Monocytes were isolated by MACS selection, pre-conditioned with cytokines for 8 h, and transduced with IDLV-G2α2pp65 for 16 h. After transduction, cells were harvested and cryopreserved at 2x106 cells/mL/vial. Cells were analyzed immediately after thaw (AT) or cultured in medium without exogenous cytokines for 7 days. (C) Viability (7AADneg) and identity (CD14 + expression level) of cell product (AT). (D) Total IDLV copy numbers detected by RT-q-PCR in the transduced cell groups AT and after 7 days in culture. (E) pp65 expression in SmyleDCpp65 (CD14neg, CD11cbright) after 7 days of in vitro culture. (F) Viability, down regulation of monocyte marker (CD14), identity (CD11cbright and HLA-DR) and functional markers (CD86 and CD80) expressed in SmyleDCpp65 7 days after in vitro culture. Additional file 2:
**Figure S2.** Cell line control used in the characterization of pp65 expression in SmyleDCpp65 (GMP-like). (A) KA2 cells and KA2 expressing pp65 (KA2/pp65) were used as negative and positive controls respectively for the qualitative determination of intracellular pp65 expression in SmyleDCpp65. Additional file 3:
**Figure S3.** Analyses of the lentiviral integration sites. RG tricistronic vector (both as integrase competent and integrase defective; MOI of 5) were used to transduce monocytes for generation of SmyleDCpp65. The cells were maintained in vitro for up to 30 days. The clonal contribution of the genetically modified monocytes was monitored with a high-throughput IS analysis. (A) Number of vector copies detected by RT-q-PCR. (B) Integration pattern of ICLV and IDLV in SmyleDCpp65 in gene versus in gene ± 10 kb. (C) Integration site frequency distribution of ICLV and IDLV in SmyleDCpp65 upstream and in genes. (D) 10 Most predominant clones for ICLV and IDLV. Colored columns represent retrieval frequency as percentage of total sequences in SmyleDCpp65 at sequential time points of analyses. Larger colored bars represent higher frequencies of integration sites clustering in the proximity of that gene. Lower panel indicate ranking of the 10 most pre-dominant clones with their corresponding color code and gene ID at sequential time points of analyses. Arrows (red) indicate recurrent insertion sites observed in the analyses.
