# Supplementary material for: Generation of lentivirus-induced dendritic cells under GMP-compliant conditions for adaptive immune reconstitution against cytomegalovirus after stem cell transplantation
Source: J Transl Med. 2015 Jul 22;13:240. doi: 10.1186/s12967-015-0599-5 (PMC4511080; doi:10.1186/s12967-015-0599-5)

Suppl Figure 1. SmyleDCpp65 validation after cryopreservation, thaw and culture

A. Integrase defective lentiviral vector backbone

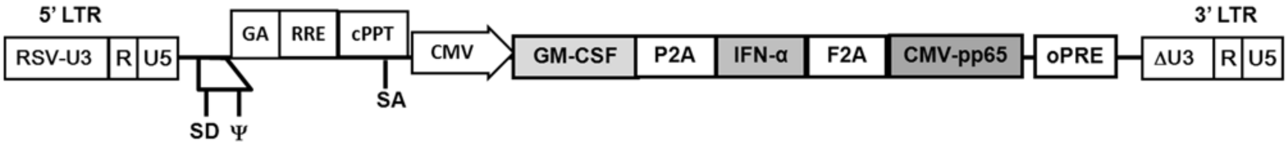

B. Scheme of SmyleDCpp65 generation

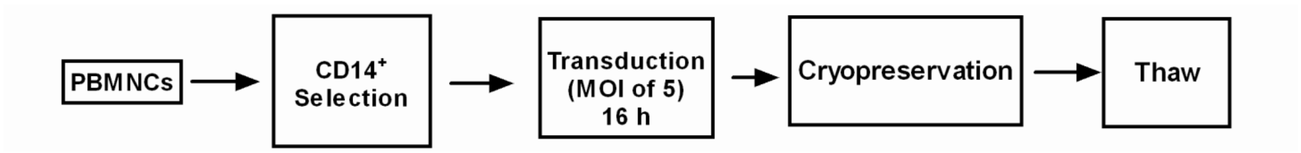

C. Viability and identity after thawing

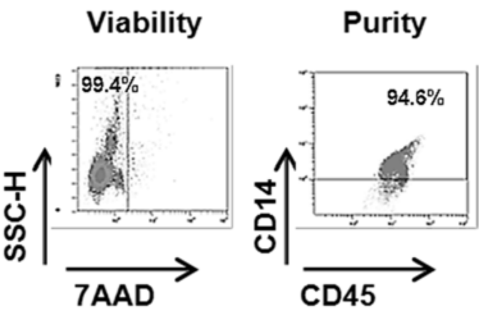

E. pp65 expression on day 7 of culture

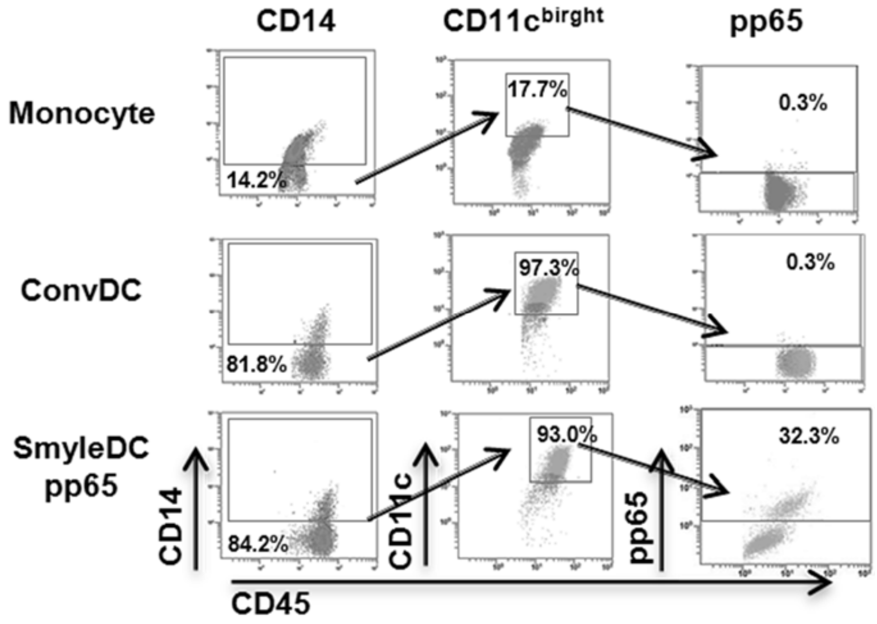

D. LV copies after thaw Vs. culture

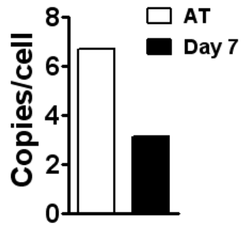

F. Immunophenotype on day 7 of culture

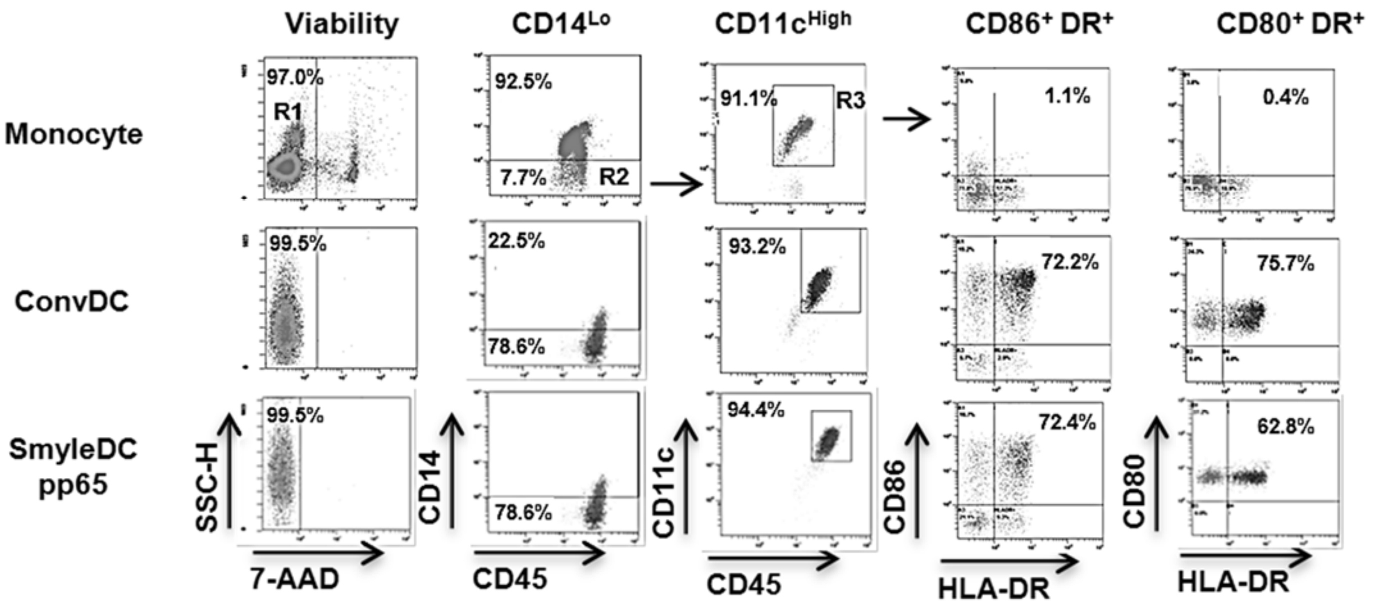

Supplement: Additional file 1: — Figure S1. Feasibility of cryopreservation. (A) Tricistronic IDLV encoding for hGM-CSF, hIFN-α and CMV-pp65 protein used to generate SmyleDCpp65. (B) Scheme of SmyleDCpp65 generation. Monocytes were isolated by MACS selection, pre-conditioned with cytokines for 8 h, and transduced with IDLV-G2α2pp65 for 16 h. After transduction, cells were harvested and cryopreserved at 2x10^6^ cells/mL/vial. Cells were analyzed immediately after thaw (AT) or cultured in medium without exogenous cytokines for 7 days. (C) Viability (7AADneg) and identity (CD14 + expression level) of cell product (AT). (D) Total IDLV copy numbers detected by RT-q-PCR in the transduced cell groups AT and after 7 days in culture. (E) pp65 expression in SmyleDCpp65 (CD14neg, CD11cbright) after 7 days of in vitro culture. (F) Viability, down regulation of monocyte marker (CD14), identity (CD11cbright and HLA-DR) and functional markers (CD86 and CD80) expressed in SmyleDCpp65 7 days after in vitro culture. [file 12967_2015_599_MOESM1_ESM.pdf]
