# Supplementary material for: Generation of lentivirus-induced dendritic cells under GMP-compliant conditions for adaptive immune reconstitution against cytomegalovirus after stem cell transplantation
Source: J Transl Med. 2015 Jul 22;13:240. doi: 10.1186/s12967-015-0599-5 (PMC4511080; doi:10.1186/s12967-015-0599-5)
Supplement: Additional file 2: — Figure S2. Cell line control used in the characterization of pp65 expression in SmyleDCpp65 (GMP-like). (A) KA2 cells and KA2 expressing pp65 (KA2/pp65) were used as negative and positive controls respectively for the qualitative determination of intracellular pp65 expression in SmyleDCpp65. [file 12967_2015_599_MOESM2_ESM.pdf]

Suppl Figure 2. Controls for flow cytometry analyses pp65 expression.

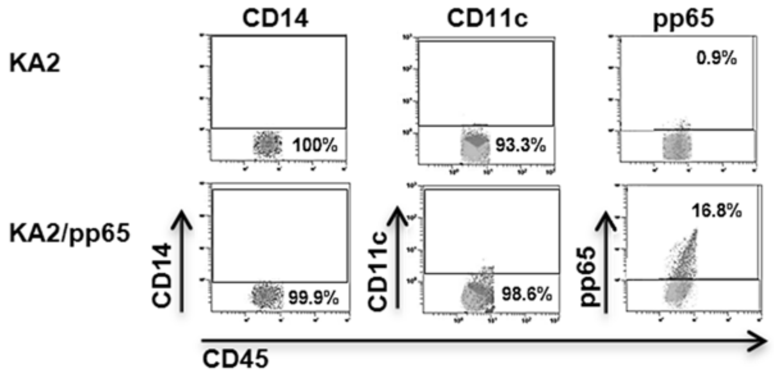

Supplementary Figure 2: Cell line control used in the characterization of pp65 expression in SmyleDCpp65 (GMP-like). (A) KA2 cells and KA2 expressing pp65 (KA2/pp65) were used as negative and positive controls respectively for the qualitative determination of intracellular pp65 expression in SmyleDCpp65.
