# Supplementary material for: Generation of lentivirus-induced dendritic cells under GMP-compliant conditions for adaptive immune reconstitution against cytomegalovirus after stem cell transplantation
Source: J Transl Med. 2015 Jul 22;13:240. doi: 10.1186/s12967-015-0599-5 (PMC4511080; doi:10.1186/s12967-015-0599-5)
Supplement: Additional file 3: — Figure S3. Analyses of the lentiviral integration sites. RG tricistronic vector (both as integrase competent and integrase defective; MOI of 5) were used to transduce monocytes for generation of SmyleDCpp65. The cells were maintained in vitro for up to 30 days. The clonal contribution of the genetically modified monocytes was monitored with a high-throughput IS analysis. (A) Number of vector copies detected by RT-q-PCR. (B) Integration pattern of ICLV and IDLV in SmyleDCpp65 in gene versus in gene ± 10 kb. (C) Integration site frequency distribution of ICLV and IDLV in SmyleDCpp65 upstream and in genes. (D) 10 Most predominant clones for ICLV and IDLV. Colored columns represent retrieval frequency as percentage of total sequences in SmyleDCpp65 at sequential time points of analyses. Larger colored bars represent higher frequencies of integration sites clustering in the proximity of that gene. Lower panel indicate ranking of the 10 most pre-dominant clones with their corresponding color code and gene ID at sequential time points of analyses. Arrows (red) indicate recurrent insertion sites observed in the analyses. [file 12967_2015_599_MOESM3_ESM.pdf]

Suppl Figure 3. Analyses of the lentiviral integration sites with research grade lentiviral vector

A. LV copies

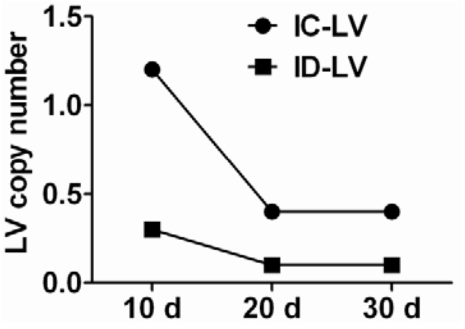

B. Vector integration in/outside gene

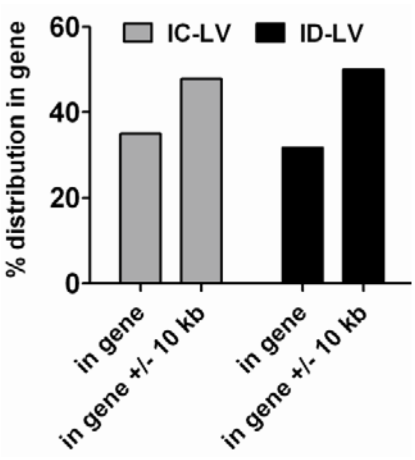

C. Distribution in gene and upstream

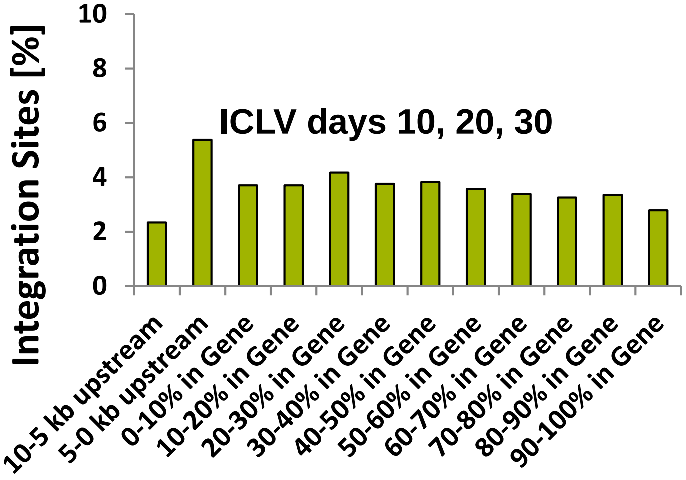

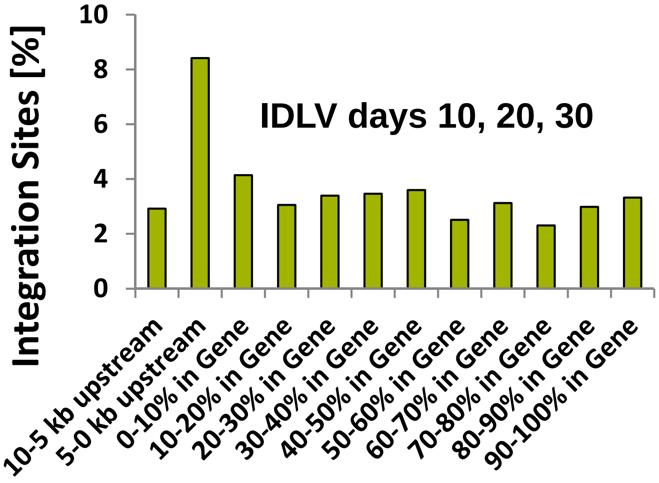

D. 10 most predominant clones)

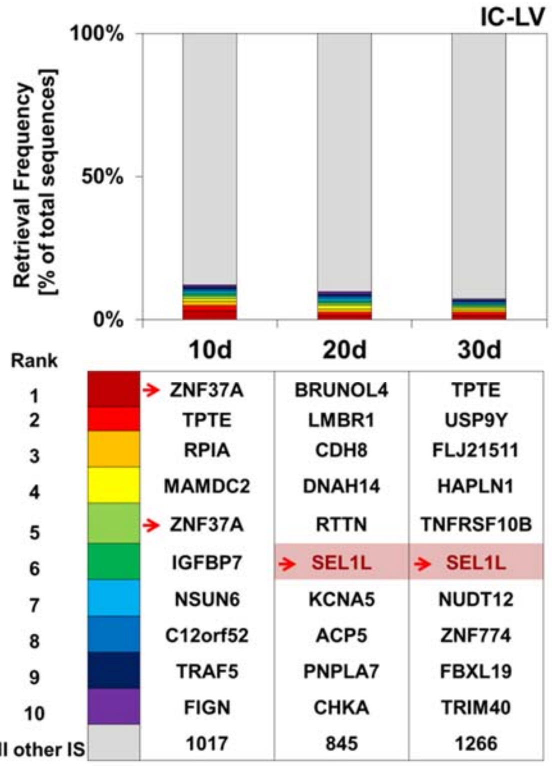

Rank	10d	20d	30d
1	→ ZNF37A	BRUNOL4	TPTE
2	TPTE	LMBR1	USP9Y
3	RPIA	CDH8	FLJ21511
4	MAMDC2	DNAH14	HAPLN1
5	→ ZNF37A	RTTN	TNFRSF10B
6	IGFBP7	→ SEL1L	→ SEL1L
7	NSUN6	KCNA5	NUDT12
8	C12orf52	ACP5	ZNF774
9	TRAF5	PNPLA7	FBXL19
10	FIGN	CHKA	TRIM40
All other IS	1017	845	1266

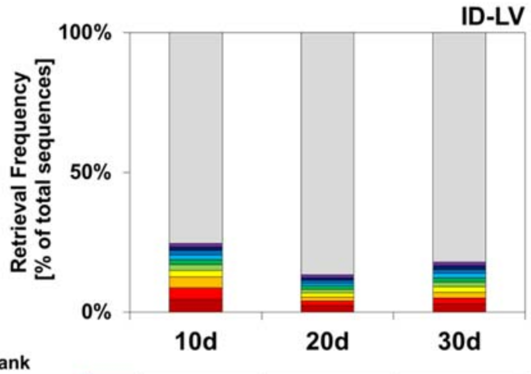

Rank	10d	20d	30d
1	→ ZNF37A	ITGAE	ALG10B
2	→ WDR74	POU3F3	SYNCRIP
3	FLJ21511	→ WDR74	FCGR1B
4	NARF	STAG3L4	ZNF33B
5	→ WDR74	CDKL5	RCOR3
6	→ MDM4	MIA3	LYRM2
7	C1orf38	ITPR2	SDCCAG1
8	→ WDR74	→ ILF2	PRIM2
9	NRXN3	INSIG1	RAB11FIP4
10	OR4F3	SLC30A5	BRD4
All other IS	408	566	469
